# Identification and Expression Analyses of SBP-Box Genes Reveal Their Involvement in Abiotic Stress and Hormone Response in Tea Plant (*Camellia sinensis*)

**DOI:** 10.3390/ijms19113404

**Published:** 2018-10-30

**Authors:** Pengjie Wang, Di Chen, Yucheng Zheng, Shan Jin, Jiangfan Yang, Naixing Ye

**Affiliations:** College of Horticulture, Key Laboratory of Tea Science, Fujian Agriculture and Forestry University, Fuzhou 350002, China; 2180311002@fafu.edu.cn (P.W.); 1160311001@fafu.edu.cn (D.C.); 1170311017@fafu.edu.cn (Y.Z.); jinshan0313@163.com (S.J.)

**Keywords:** *Camellia sinensis*, SBP-box, abiotic stress, hormone, expression profile

## Abstract

The *SQUAMOSA* promoter binding protein (SBP)-box gene family is a plant-specific transcription factor family. This family plays a crucial role in plant growth and development. In this study, 20 SBP-box genes were identified in the tea plant genome and classified into six groups. The genes in each group shared similar exon-intron structures and motif positions. Expression pattern analyses in five different tissues demonstrated that expression in the buds and leaves was higher than that in other tissues. The *cis*-elements and expression patterns of the *CsSBP* genes suggested that the *CsSBP* genes play active roles in abiotic stress responses; these responses may depend on the abscisic acid (ABA), gibberellic acid (GA), and methyl jasmonate (MeJA) signaling pathways. Our work provides a comprehensive understanding of the *CsSBP* family and will aid in genetically improving tea plants.

## 1. Introduction

Tea is the oldest natural, nonalcoholic, caffeine-containing beverage and benefits human health due to its wealth of secondary metabolites, including catechins, theanine, polysaccharides, caffeine, and volatiles [1]. The tea plant, *Camellia sinensis* (L.) O. Kuntze, is an economically valuable perennial evergreen crop that has been widely cultivated in more than 100 countries [2]. In many areas, tea plants are continuously challenged by a wide variety of abiotic stresses, such as low temperature, drought, and salinity [3,4]. One or a combination of several environmental stresses can lead to substantial losses in tea production and reduced tea quality [5].

In higher plants, transcription factors (TFs) play critical roles in the regulation of physiological processes and adaptation to environmental stresses through various signal transduction pathways [6]. *SQUAMOSA* promoter binding protein (SBP)-box genes encode a plant-specific family of TFs that have a highly conserved SBP domain of approximately 76 amino acid residues. This conserved domain comprises three functionally important motifs, including two zinc-finger structures and a bipartite nuclear localization signal (NLS) that partially overlaps with the second zinc-finger [7]. Two *SBP* genes (*AmSBP1* and *AmSBP2*) were first isolated from *Antirrhinum majus* based on their direct interaction with a region in the promoter of the floral meristem identity gene *SQUAMOSA* [8]. The *AtSPL3* gene was the first *SBP* gene identified in *Arabidopsis*, where it plays a pivotal role in promoting flowering under long-day conditions. In addition, *AtSPL3* recognizes a conserved element in the promoter region of *APETALA1* (AP1), an ortholog of *SQUAMOSA* [9].

Previous studies have indicated that the *SBP* genes play essential role in diverse developmental processes in plants, such as sporogenesis [10], shoot and leaf development [11,12], flowering [13], fertility [14], fruit ripening [15], plant hormone signal transduction [16,17], and copper homeostasis [18]. Moreover, *OsSPL14* and *OsSPL16* were reported to promote grain quality and yield in rice [19,20]. Recent studies have shown that the *SBP* genes are involved in hormone signal transduction and responses to abiotic and biotic stress in many plant species. For example, *VpSBP16* overexpression enhances salt and drought stress tolerance during seed germination, as well in seedlings and mature plants, by regulating the salt overly sensitive (SOS) and reactive oxygen species (ROS) signaling cascades in transgenic *Arabidopsis* [21]. Co-expression analysis revealed that the expression of *AtSPL* genes is significantly correlated with that of genes involved in the defense response pathway in response to various biotic and abiotic stresses [22]. *Arabidopsis AtSPL14* has been found to be involved in the development of normal plant architecture and to play a role in sensitivity to the fungal toxin fumonisin *B1* [23]. At5g43270 (*AtSPL2*) is modified in transgenic *Arabidopsis* overexpressing the *AtJMT* gene in response to the jasmonic acid (JA)-mediated resistance pathway [24]. The *BpSPL9* gene in birch was reported to increase resistance to abiotic stress by increasing the accumulation of superoxide dismutase (SOD) and peroxidase (POD) in transgenic lines [25]. Additionally, the preliminary expression profiles of 31 maize *SBP* genes indicated that some were affected by several stress stimuli, including cold, drought, salinity, and abscisic acid (ABA) exposure [26].

With the rapid development of next-generation sequencing technologies, SBP-box gene families have been identified in many plant species to date, including 16 *SBP* genes in *Arabidopsis* [27], 19 in rice [28], 18 in grape [29], 12 in birch [25], 16 in jujube [30], 32 in moso bamboo [31], and 27 in apple [32]. However, no systematic identification or characterization of *SBPs* has been conducted in tea plants to find indications of candidate *SBP* genes involved in responses to stress and hormones. Tea plant genome sequences (vars. assamica and sinensis) [33,34] completed and released in 2017 and 2018 can provide an opportunity to identify all tea plant SBP genes. In the present study, we conducted the genome-wide identification of *CsSBP* genes in tea plants and analyzed their classification, structure, conserved motifs, *cis*-elements, and expression patterns in five different tissues. Furthermore, we investigated the expression profiles of *CsSBP* genes in response to several abiotic stresses and plant hormones to determine their potential functions in stress tolerance. These results will facilitate the further exploration of *CsSBP* gene functions and responses to environmental stress in tea plants.

## 2. Results

### 2.1. Identification of CsSBP Gene Family Members in Tea Plants

**Figure 1:**
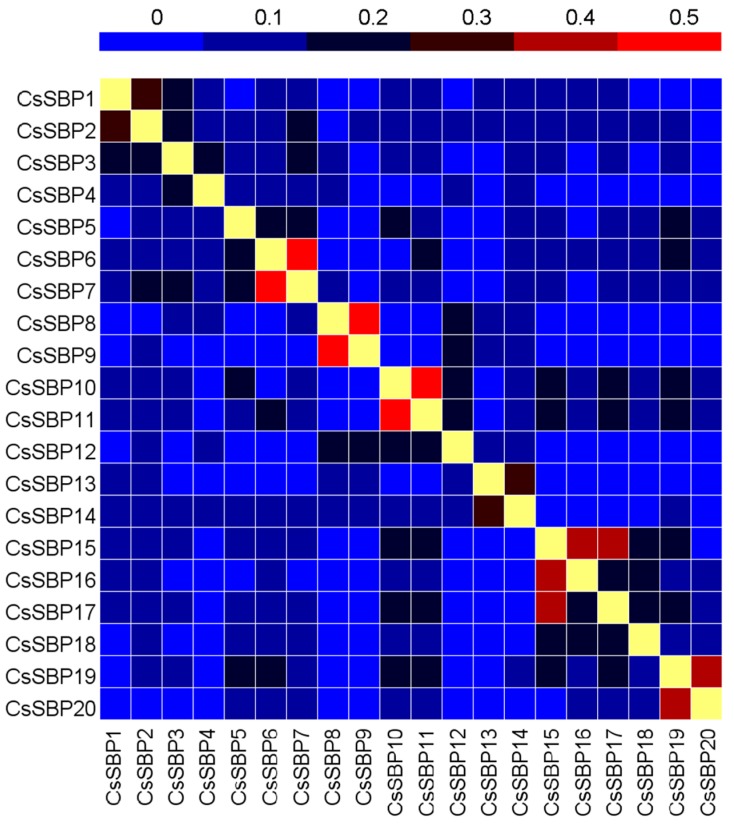
The percentage identity matrix of CsSBP proteins. The heatmap was conducted based on the CsSBP protein sequences. The colored bar indicates the correlation of protein sequences of two genes, blue represents a low correlation, red represents a high correlation, and yellow represents a correlation that is 1. The correlated values of the CsSBP proteins are listed in Table S2.

In this study, 20 SBP-box genes were identified in the tea plant genome. We named the 20 SBP-box genes *CsSBP1* to *CsSBP20* based on their phylogenetic distance. The details of the *CsSBP* gene family in tea plant are given in Table 1 and Table S1. The lengths of the genomic sequences ranged from 373 bp (*CsSBP16*) to 9742 bp (*CsSBP13*), and the average was 3920 bp. The coding sequences (CDS) of the *CsSBP* genes varied from 338 bp (*CsSBP16*) to 3399 bp (*CsSBP16*) and had predicted proteins of 110–1132 amino acids, which is consistent with the length of *AtSPL* genes from *Arabidopsis* widely ranging from 396 (*AtSPL3*) to 3108 bp (*AtSPL14*) with the deduced proteins of 131 to 1035 amino acids. The molecular weight (MW) of CsSBP proteins ranged from 11.60 (CsSBP12) to 124.64 (CsSBP8) kDa, the isoelectric point (pI) ranged from 5.60 (CsSBP13) to 9.14 (CsSBP19), indicating that 13 CsSBP proteins were basic proteins with pI value more than 7.0, and the remaining seven proteins were acidic proteins. The percentage identity matrix of CsSBP proteins based on the sequence alignment is listed in Figure 1 and Table S2. CsSBP10 and CsSBP11, CsSBP8 and CsSB9, CsSBP6, and CsSBP7 share the highest identity 70%, 68%, and 56%, respectively. Additionally, most CsSBP proteins have low identity to each other, suggesting the diversity of this family during evolution.

### 2.2. Sequence Alignments and Phylogenetic Analysis of SBP Domains

**Figure 2:**
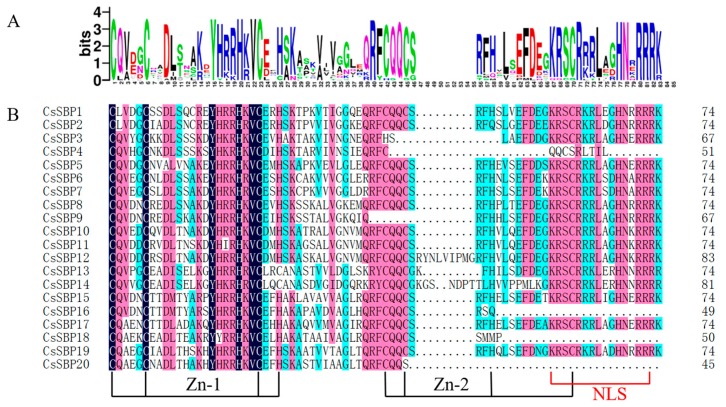
Multiple sequence alignment of the *SQUAMOSA* promoter binding protein (SBP) domains in tea plant. (**A**) The sequence logos of the conserved SBP domains were analyzed using WebLogo. The *y* axis (measured in bits) depicts the overall height of the stack, indicating the sequence conservation at that position, while the height of the symbols within the stack indicates the relative frequency of each amino at that position. (**B**) The SBP domain sequences were aligned using DNAMAN 7.0 software; the two zinc-finger structures and nuclear localization signal (NLS) are indicated.

To determine the SBP domains in each CsSBP protein, we performed sequence alignment using the DNAMAN 7.0 program (Figure 2). All SBP domains from the CsSBP proteins contain two zinc-finger structures (C3H, C2HC) and a bipartite nuclear localization signal, with the exceptions of CsSBP3, CsSBP4, CsSBP9, CsSBP16, CsSBP18, and CsSBP20, which lack the C2HC or NLS (Figure 2B). Analysis by NLS Mapper online software found that this CsSBP proteins did not have other possible high-confidence NLSs. Furthermore, we predicted the subcellular localization of these proteins and found that these proteins were also most likely to be located in the nucleus (Table S3). Interestingly, most SBP domains were consecutive and lacked insertions; the exceptions were CsSBP12 and CsSBP14, whose SBP domains were interrupted by a short sequence. Furthermore, the sequence logos of these SBP domain sequences showed that some positions were highly conserved (Figure 2A); these included YHRRHKVC, CQQC, KRSCR, and RRR sequences.

**Figure 3:**
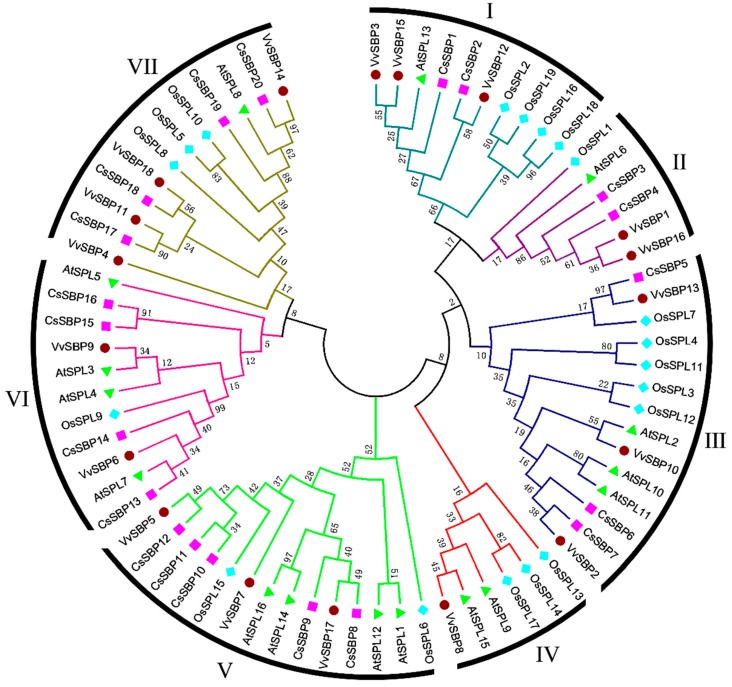
Phylogenetic tree of SBP domains created in MEGA 5.0. The sequences of the SBP domain are from tea plant (CsSBP), *Arabidopsis* (AtSPL), rice (OsSPL) and grape (VvSBP). All SBP members were classified into seven groups (I–VII), and their sequences and sources are listed in Table S4.

To investigate the molecular evolution of and phylogenetic relationships among SBP genes in plants, 73 conserved SBP domain sequences from four species were used to construct a phylogenetic tree (Table S4); the tree included 19 sequences from a monocotyledonous angiosperm (rice) and 54 sequences from dicotyledonous angiosperms (*Arabidopsis*, grape and tea plant). As shown in Figure 3, all SBP-box genes were able to be categorized into seven groups (I–VII). Each group, except for group IV, contained at least one SBP member from each of the four species; group IV lacked SBP members from tea plant. Groups III and V composed the largest clade, which included fourteen members and almost 19% of all *SBP* genes, while groups II and IV had the smallest clade, which contained only six members. Additionally, all of the *OsSPL* genes in each group were very dissimilar compared to those of other dicotyledons, and most of the *CsSBP* genes were closer to those of grape.

### 2.3. Structural Organization and Conserved Motif Analysis of CsSBP Genes

**Figure 4:**
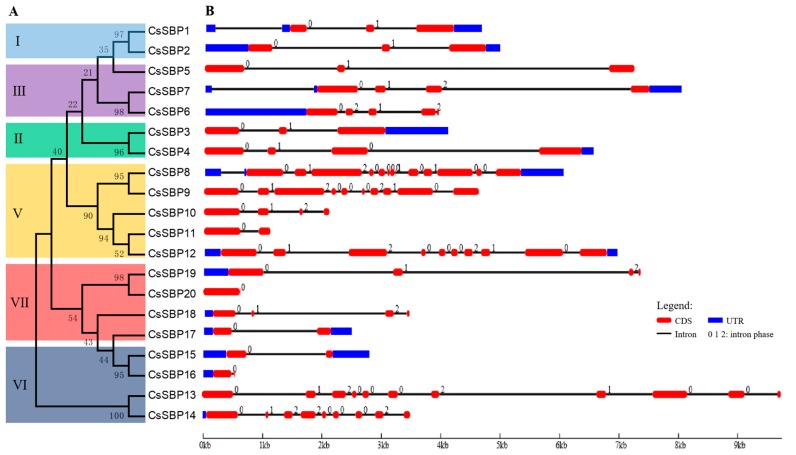
Phylogenetic tree of the *CsSBP* gene family and the exon-intron structures of tea plant SBP proteins. (**A**) The phylogenetic tree of the *CsSBP* genes was created using the MEGA 5.0 software with the neighbor-joining method; bootstrap values from 1000 replicates are indicated at each node. (**B**) Exon-intron structures were described using GSDS 2.0. Red, round-cornered rectangles represent exons, black lines represent introns, and blue rectangles represent untranslated regions (UTRs). The numbers 0, 1, and 2 represent intron phases.

To assess the structural diversity among the six groups, we constructed a phylogenetic tree using the predicted full-length CsSBP protein sequences (Figure 4A). The exon-intron structures of all 20 *CsSBP* genes were generated based on their corresponding coding and genome sequences (Figure 4B). The number of exons in the *CsSBP* genes varied from one (*CsSBP20*) to twelve (*CsSBP8*), with an average of 4.85 introns per *CsSBP* gene. Additionally, many *CsSBP* genes had two to four exons: 4 out of 20 had two exons, 4 had three exons, and 6 had four exons. In general, most *CsSBP* genes had exon structures similar to those of the common group, with some exceptions, such as *CsSBP10* and *CsSBP11* in group V (Figure 4B). For example, the *CsSBP* genes within group I had three exons, whereas those within group III contained more than three exons. It is notable that the CDS lengths and gene structures of some *CsSBP* gene pairs were quite similar, including *CsSBP1* and *CsSBP2*, *CsSBP3* and *CsSBP4*, *CsSBP9* and *CsSBP12*, *CsSBP13* and *CsSBP14*, *CsSBP18* and *CsSBP19* (Figure 4B), suggesting that these gene pairs may be tandem duplicated gene pairs within the tea plant *CsSBP* genes. However, due to the lack of chromosomal localization information in the tea plant genome, no further analysis can be performed.

**Figure 5:**
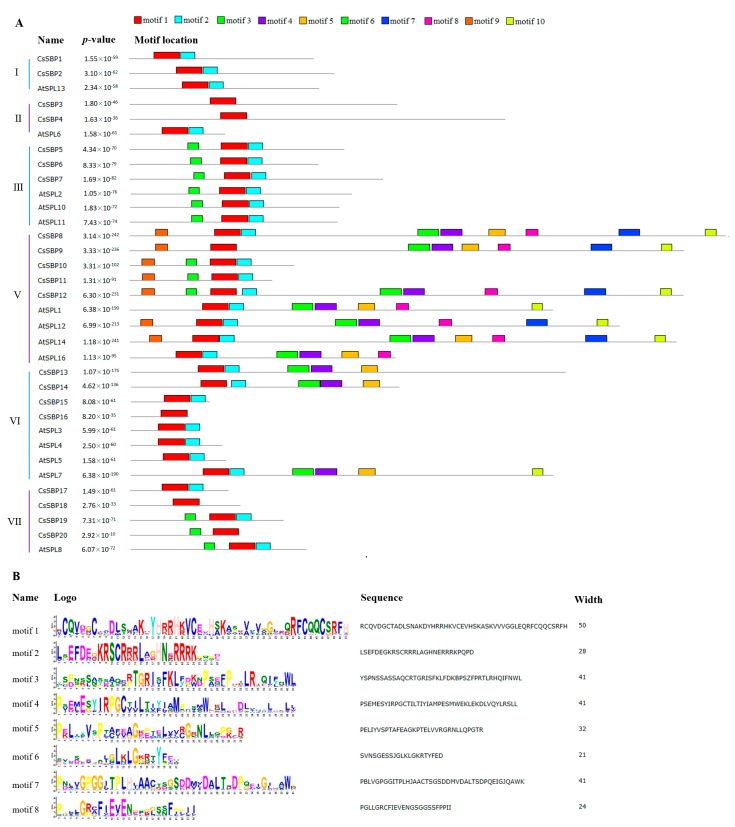
Conserved motifs of CsSBP proteins from tea plant and *Arabidopsis*. (**A**) The different-colored boxes represent different motifs and their positions in each SBP sequence. (**B**) The sequences and logos of the identified motifs in tea plant and *Arabidopsis*.

We further searched for the presence of conserved motifs in the 20 CsSBP proteins using MEME software compared to *Arabidopsis*, and ten most conserved motif were presented and designated motif 1 to motif 10 (Figure 5A). The details of the conserved motifs and their lengths are shown in Figure 5B. Motif 1 and motif 2 represent the SBP domains and were present in all SBP sequences; however, six SBP domains (CsSBP3, CsSBP4, CsSBP9, CsSBP16, CsSBP18 and CsSBP20) were incomplete (Figure 5A). Furthermore, motif 8 consisted of an ankyrin-repeat-containing domain and was found exclusively in three CsSBP proteins (CsSBP8, CsSBP9 and CsSBP12) and four AtSPL proteins (AtSPL1, AtSPL12, AtSPL14 and AtSPL16) from group V, indicating that these CsSBP proteins may interact with other proteins in their functions in plant cells [35]. Members of the same group usually had similar motif compositions, and some motifs were specifically present in one or more groups. For example, the members of group I only contained motifs 1 and 2, and the locations of the motifs were similar. Motifs 7, 8 and 9 were unique to group V. Four motifs (motifs 3, 4, 5 and 10) were exclusively present in groups V and VI (Figure 5A).

### 2.4. cis-Element Analysis of CsSBP Genes

**Figure 6:**
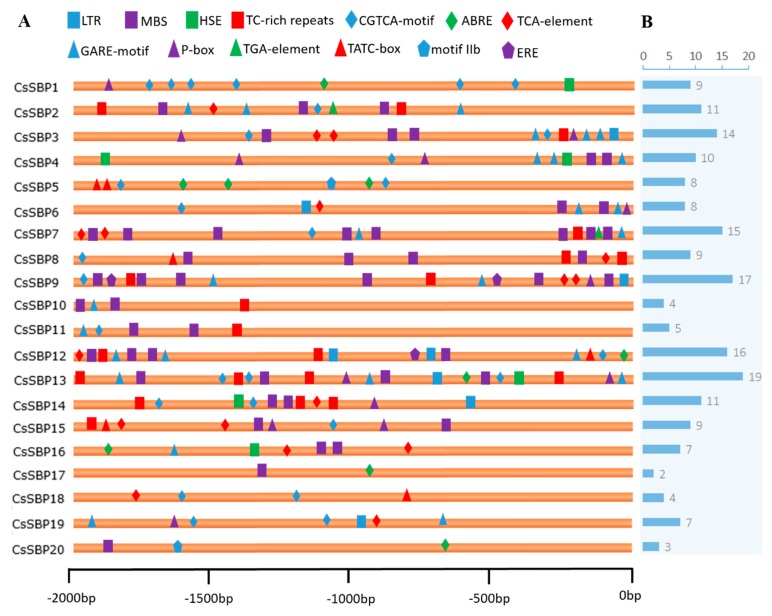
Predicted *cis*-elements associated with various stress and hormone responses in the *CsSBP* promoters. (**A**) Depiction of the distribution of *cis*-elements that relate to various stress and hormone responses in the 2 kb upstream promoter regions of *CsSBP* genes. Different *cis*-elements are represented by different shapes and colors. (**B**) Numbers of stress- and hormone-response-related *cis*-elements in each *CsSBP* promoter.

The analysis of *cis*-elements in promoter sequences is extremely important for understanding gene regulation and function. Thirteen types of stress- and hormone-related *cis*-elements were identified in the 2000 bp promoter sequences of the *CsSBP* genes (Figure 6A). These *cis*-elements included low-temperature-responsive element (LTR), MYB binding sites involved in drought inducibility (MBS), heat-stress-responsive element (HSE), defense- and stress-responsive elements (TC-rich repeats), MeJA-responsive element (CGTCA-motif), abscisic acid-responsive element (ABRE and motif IIb), salicylic-acid-responsive element (TCA-element), gibberellin-responsive element (GARE motif, P-box, and TATC-box), auxin-responsive element (TGA-element), and ethylene-responsive element (ERE) (Figure 6A). All *CsSBP* promoter sequences possessed stress- and hormone-associated *cis*-elements, but the number varied from 2 (*CsSBP17*) to 19 (*CsSBP13*) (Figure 6B). In total, 48 MBS were found in 16 (80%) *CsSBP* promoter sequences, composing the largest proportion of all elements and suggesting a potential role in drought inducibility. In addition, low-temperature-, heat-, ABA-, MeJA-, GA-, and SA-responsive elements were abundant in the promoter sequences of the *CsSBP* genes, suggesting that these genes may be involved in the transcriptional control of abiotic stress and hormone responses.

### 2.5. Expression Patterns of CsSBP Genes in Different Tissues Based on Transcriptome Data

**Figure 7:**
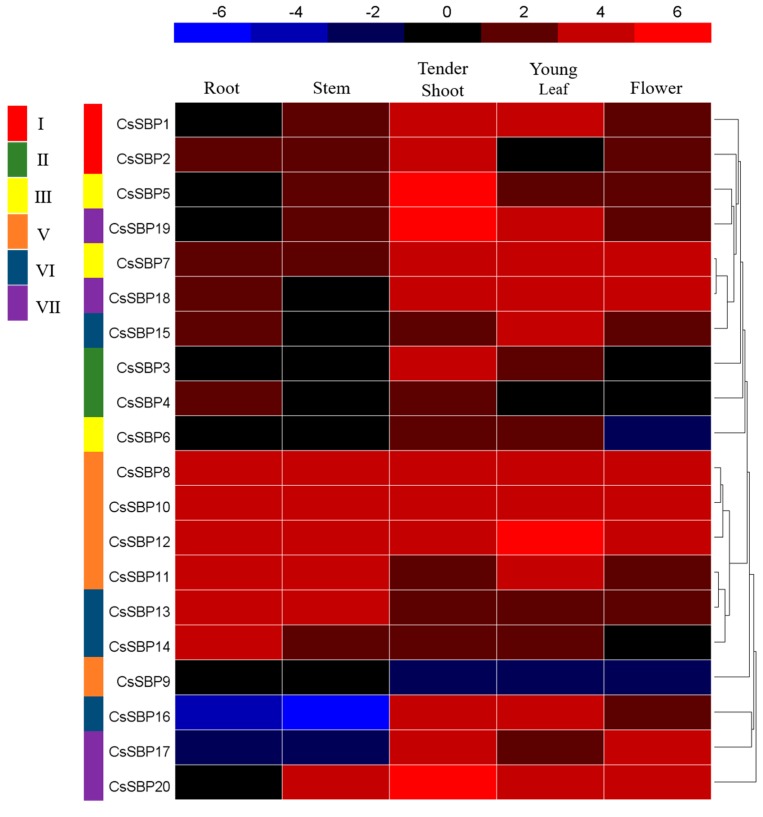
Expression profiles of *CsSBP* genes in different tissues. The colored bar represents the expression values [log_2_(FPKM)]. Blue represents low expression, and red represents high expression. Different groups of *CsSBP* genes are marked with rectangles of different colors. The FPKM values of the *CsSBP* genes in different tissues are listed in Table S5.

The transcriptomes of five tea plant tissues are available online and were downloaded to investigate the expression patterns of *CsSBP* genes (Figure 7). The fragments per kilobase million (FPKM) values of the *CsSBP* genes in different tissues are listed in Table S5. All *CsSBP* genes were expressed in the five tested tissues at diverse expression levels; the exception was *CsSBP16* in the stem. The transcripts of *CsSBP7*, *CsSBP8*, *CsSBP10*, *CsSBP11*, *CsSBP12*, and *CsSBP13* were highly expressed (value > 2) in these five tissues, suggesting that *CsSBP* genes play essential roles in plant growth and development. Several *CsSBP* genes exhibited a degree of tissue specificity. For instance, *CsSBP16* and *CsSBP17* were only highly expressed (value > 2) in tender shoots, young leaves, and flowers, while the transcripts of *CsSBP3* and *CsSBP6* largely accumulated in tender shoots and young leave rather than in other tissues. In general, the expression of *CsSBP* genes was highest in the tender shoots, followed by the young leaves, flowers, roots, and stems. This expression pattern implied that *CsSBP* genes might have important functions in the buds and leaves.

### 2.6. Expression Patterns of CsSBP Genes under Cold, Drought, ABA, GA, and MeJA Treatment as Evaluated by qRT-PCR

**Figure 8:**
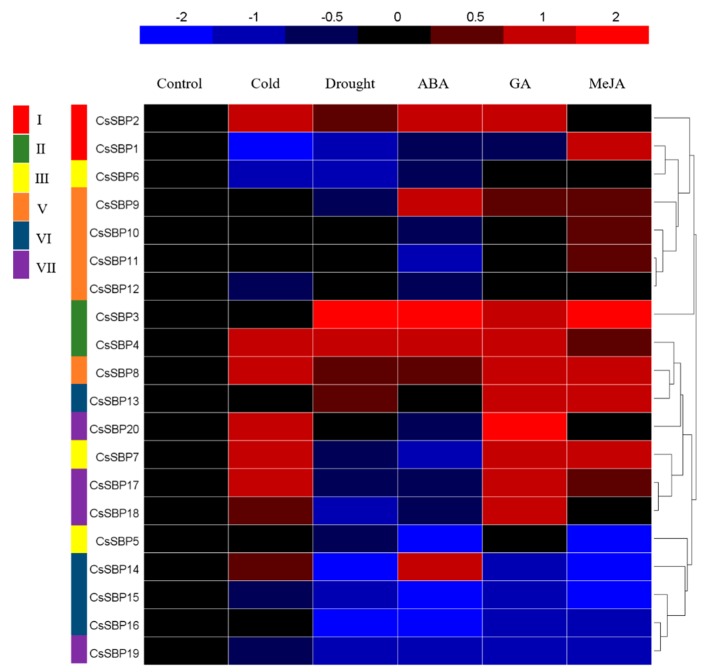
Expression profiles of *CsSBP* genes under cold, drought, ABA, GA, and MeJA treatments as determined by qRT-PCR. The relative gene expression levels were calculated using the 2^−ΔΔ*C*t^ method and are expressed as the fold change relative to the expression of the control. Three independent biological replications were performed. The colored bar represents Log_2_ expression values. Blue represents low expression and red represents high expression. Different groups of *CsSBP* genes are marked with rectangles of different colors. The expression value of *CsSBP* genes under five treatments are listed in Table S6.

To investigate the potential role of *CsSBP* genes in the responses to various abiotic stresses and hormones, qRT-PCR was used to analyze the expression of 20 *CsSBPs* under cold, drought, ABA, GA, and MeJA treatment, and the expression values under five treatments are listed in Table S6. A heat map based on their expression levels is shown in Figure 8. *CsSBPs* with normalized expression values ≥0.5 were considered up-regulated genes, and those with values ≤−0.5 were defined as down-regulated. Overall, compared to their expression in the control (untreated group), the expression of the 20 *CsSBP* genes was significantly affected by these treatments, implying that these genes may have multiple functions in the response to stress stimuli. In detail, eight (*CsSBP2*, *CsSBP4*, *CsSBP7*, *CsSBP8*, *CsSBP14*, *CsSBP17*, *CsSBP18*, and *CsSBP20*) and five (*CsSBP2*, *CsSBP3*, *CsSBP4*, *CsSBP8*, and *CsSBP13*) *CsSBP* genes were significantly up-regulated under cold and drought stresses, respectively (Figure 8). Furthermore, 11, 14, and 15 genes were found to potentially be affected by ABA, GA, and MeJA, respectively. For these treatments, the most up-regulated gene was *CsSBP3*, while the most down-regulated gene was *CsSBP15*. Stress-specific responses were also found; the transcripts of three genes (*CsSBP1*, *CsSBP10*, and *CsSBP11*) were significantly elevated only under MeJA treatment. Several genes, such as *CsSBP5*, *CsSBP15*, *CsSBP16*, and *CsSBP19*, were generally down-regulated under these treatments.

## 3. Discussion

Because of the active role of SBP-box genes in plant growth and environmental responses, many SBP-box families have been identified in various plant species based on whole-genome sequencing [27,28,29]. However, there have been no studies about the *SBP* genes in tea plants. In this study, we conducted comprehensive research on this important transcription factor family in tea plants, including the identification of *CsSBP* gene family members and the analysis of their physical and chemical parameters, their classification, exon-intron structure, conserved motifs, *cis*-elements, expression patterns in different tissues, and their responses to various stresses and hormones.

A total of 20 *CsSBP* genes were identified in the tea plant genome. There were 16, 19, 18, and 12 *SBP* members in *Arabidopsis* [27], rice [28], grape [29], and birch [25], respectively. The number of *CsSBPs* is greater than that in these plant species but slightly less than that in petunia [36] (21 *SBPs*) and apple [32] (27 *SBPs*). Although the tea plant genomes (3.14 Gb and 3.02 Gb) are much larger than those of other plants, such as *Arabidopsis* [37] (genome size 125 Mb) and rice [38] (390.3 Mb), many studies have confirmed that this size resulted from the long-term amplification of a few LTR retrotransposon families [33,34]. The SBP-box family TFs from tea plant clustered into six groups (Figure 3), but not group IV, which is found in other plants, perhaps because of their ancient and divergent evolutionary patterns [39]. Moreover, this classification was generally consistent with gene structures and motif localizations (Figure 4 and Figure 5) as described previously in rice and apple [28,32]. However, some motifs were found in specific groups. For example, motifs 7, 8, and 9 existed only in group V (Figure 5). These motifs may be associated with the functional diversity of the SBP family.

To further explore the potential functions of the *CsSBP* family in tea plant growth and development, the expression patterns of the 20 *CsSBP* members in five different tissues were examined based on published transcriptome data (Figure 7). Our results indicated that most *CsSBP* genes are highly and widely expressed in the different tissues we tested. The transcripts of six members (*CsSBP7*, *CsSBP-8*, *CsSBP10*, *CsSBP11*, *CsSBP12*, and *CsSBP13*) accumulated to high levels in all tissues, suggesting essential roles. Interestingly, the expression of *CsSBP* genes in the buds and leaves was higher than that in other tissues. The tender shoots and young leaves are the most economically important tissues and are processed into beverages [40]. Homologous genes of *CsSBP6* and *CsSBP7* include *AtSPL2*, *AtSPL10,* and *AtSPL11*, which have been reported to affect leaf shape and shoot maturation [41]. The high expression of *CsSBP6* and *CsSBP7* in shoots and leaves suggests that these genes may play vital roles in shoot and leaf development. Furthermore, *AtSPL14* in *Arabidopsis*, which is an ortholog of *CsSBP8* and *CsSBP9*, is quite active in the development of normal plant architecture [23]. *CsSBP1* exhibited high expression levels in tender shoots and clustered closely with *AtSPL13* in the phylogenetic tree, while *AtSPL13* is predominantly expressed in the hypocotyl and affects leaf primordium development [42]. *CsSBP17* was highly expressed in flowers, consistent with the function of its grape ortholog *VvSBP11* [43]. Overall, our expression data on *CsSBP* genes in different tissues showed high expression in shoots and will aid in further functional analysis of *SBP* members in tea plants.

Although several physiological developmental processes regulated by *SBP* genes have been discovered in recent years, only a few *SBP* genes have been shown to act under stress. For instance, combined analyses of co-expression networks and promoters have revealed that *AtSPL* genes are significantly correlated with genes involved in the defense pathway in response to various biotic and abiotic stresses [22]. *Arabidopsis AtSPL14* has been reported to be involved in programmed cell death and to play a role in sensitivity to the fungal toxin fumonisin *B1* [23]. Moreover, the *BpSPL9* gene in birch has been demonstrated to increase the resistance of transgenic lines to abiotic stress [25]. In tea plants, we found that eight (*CsSBP2*, *CsSBP4*, *CsSBP7*, *CsSBP8*, *CsSBP14*, *CsSBP17*, *CsSBP18*, and *CsSBP20*) and five (*CsSBP2*, *CsSBP3*, *CsSBP4*, *CsSBP8*, and *CsSBP13*) *CsSBP* genes were obviously up-regulated under cold and drought stress, respectively (Figure 8). The grape gene *VvSBP16* is an ortholog of *CsSBP3* and *CsSBP4* and enhances tolerance to drought stress by regulating the SOS and ROS signaling cascades [21]. In addition, we observed many stress-related *cis*-elements in the upstream regulatory sequences of *CsSBP* genes, especially drought- and low-temperature-related *cis*-elements (Figure 6), which suggested the involvement of *CsSBP* family members in abiotic stress. Numerous studies have shown that stress responses are regulated by signal transduction pathways, such as those for the hormonal signals ABA, GA, salicylic acid (SA), and MeJA, which can interact to activate the transcription of diverse stress-related genes [44,45]. Through a comprehensive analysis of the promoter regions of the *CsSBP* genes, we found numerous hormonal-response-related *cis*-elements, particularly many ABA-, GA-, and MeJA-responsive *cis*-elements (Figure 6). Therefore, we investigated the expression profiles of the *CsSBP* family under treatment with these three hormones. The results indicated that 12, 14, and 15 of the 20 *CsSBP* genes were regulated by ABA, GA, and MeJA, respectively. Furthermore, we found that members of the same group have similar gene expression patterns, especially group II, group V, and group VI (Figure 7 and Figure 8). Most of the *CsSBP* genes that were significantly induced by drought, GA, and MeJA treatments contained corresponding *cis*-elements: 9 of the 12 members significantly induced by drought stress contained corresponding MBS *cis*-elements; 12 of the 14 members significantly induced by GA treatment contained the corresponding GARE-motif, P-box and TATC-box elements; 12 of the 15 members significantly induced by MeJA contained the corresponding CGTCA *cis*-elements; suggesting the important role of these *cis*-elements involved in plant regulatory networks control. In summary, our studies suggest that the *CsSBP* genes have multiple functions in tea plant and play active roles in responses to abiotic stresses, possibly by relying on the ABA, GA, and MeJA signaling pathways.

## 4. Materials and Methods

### 4.1. Database Searches for CsSBP Gene Family Members in Tea Plants

Hidden Markov model (HMM) profiles of all sequences containing an SBP domain (PF03110) were used to search the Tea plant Genome Database (http://www.plantkingdomgdb.com/tea_tree/) [33,46]. To verify the putative *CsSBP* genes, SMARAT (http://smart.embl-heidelberg.de/) and CCD (https://www.ncbi.nlm.nih.gov/cdd/) were used to confirm the existence of the conserved SBP domain. Information on the *CsSBP* genes and predicted proteins, including the lengths of their genomic sequences, coding sequences (CDS), and open reading frames (ORFs), was obtained from the Tea Plant genome Database. Building a percentage identity matrix of CsSBP proteins based on the sequence alignment using DNAMAN 7.0 software and and presented as heat map in HemI 1.0 software. ExPASy (http://www.expasy.org/) was used to calculate the physical and chemical parameters of the CsSBP proteins. The NLS mapper (http://nls-mapper.iab.keio.ac.jp/cgi-bin/NLS_Mapper_form.cgi) and WoLF PSORT programs (https://wolfpsort.hgc.jp/) were used to analyze the NLS and subcellular localization information of the CsSBP protein, respectively. 

### 4.2. Sequence Alignment and Phylogenetic Analysis

The sequences of the CsSBP proteins obtained from the tea plant genome and their conserved domains were aligned using DNAMAN 7.0 software. The conserved domains were also analyzed using the online platform WebLogo (http://weblogo.berkeley.edu/logo.cgi) [47]. For better classification, a phylogenetic tree was constructed using MEGA 5.0 (https://www.megasoftware.net/index.php) with the neighbor-joining method and 1000 bootstrap replicates based on the conserved SBP domains and the full-length sequences [48].

### 4.3. Gene Structure and Conserved Motif Analysis

The structures of the *CsSBP* genes were obtained from gene annotation files (http://www.plantkingdomgdb.com/tea_tree/data/gff3/), and the graph of the exon-intron structures was generated using the web-based Gene Structure Display Server (http://gsds.cbi.pku.edu.cn/) [49]. The full-length sequences of the SBP proteins from tea plant and *Arabidopsis* were analyzed using the MEME online tool (http://meme-suite.org/tools/meme) to predict conserved motifs. The numbers of motif was set to ten [50].

### 4.4. Putative Promoter Region Analysis

The *cis*-acting elements in the putative promoter region were investigated using the online website PlantCARE (http://bioinformatics.psb.ugent.be/webtools/plantcare/html/), and the upstream genomic sequence within 2000 bp of the start codon of each *CsSBP* gene was analyzed [51].

### 4.5. Expression Profile Analysis Based on RNA-Seq Data

The raw transcriptomic data from different tissues, including roots, stems, tender shoots, young leaves, and flowers (SRP103063), were downloaded from the NCBI Sequence Read Archive (SRA) website reported previously by Xia et al. [33]. All available reads were mapped to the tea plant genome using TopHat2 version 2.08 with default parameters [52]. HTseq software version 0.9.1 was used to quantify gene expression levels based on FPKM values [53]. The expression profiles were incorporated into heat maps using HemI 1.0 software.

### 4.6. Tea Plant Materials and Treatments

The experimental materials in this study were three-year-old seedlings grown from cuttings of the cultivar ‘Tieguanyin’ and were cultivated in the plant germplasm collection garden at Fujian Agriculture and Forestry University, Fuzhou, Fujian Province, China. For the low-temperature treatment, tea plants grown under ambient temperatures of 24 ± 2 °C were transferred to a growth chamber at 4 °C. For drought treatment, tea plants were transferred to a 10% (*w*/*v*) PEG-6000 solution. For the ABA, methyl jasmonate (MeJA) and gibberellic acid (GA) treatments, a freshly prepared working solution of 100 µM ABA, MeJA, or GA was sprayed on the tea leaves. The second leaves of the plants from the five treatments and from control (untreated) plants were collected after 12 h. Three independent biological replicates were collected, frozen in liquid nitrogen and stored at −80 °C.

### 4.7. RNA Extraction and qRT-PCR Analysis

Total RNA from second leaves was extracted using an RNAprep Pure Plant kit (DP441, TIANGEN, Beijing, China) in accordance with the manufacturer’s instructions. An EasyScript One-Step gDNA Removal and cDNA Synthesis SuperMix kit (AE311-02, TransGen Biotech, Beijing, China) was used to reverse transcribe the total RNA to cDNA for fluorescence detection. Quantitative real-time PCR (qRT-PCR) was conducted on a CFX96 Touch™ Real-Time PCR detection system (Bio-Rad, Hercules, CA, USA) using SYBR Green. The housekeeping gene *GAPDH* (accession no. GE651107) was used as an internal control. Gene-specific primers for the *CsSBP* genes were designed using Primer Premier 6.0 (Table S7). The reactions were conducted according to the method provided with the TransStart^®^ Tip Green qPCR SuperMix kit (AQ141-02, TransGen Biotech, Beijing, China) using the following procedure: 95 °C for 30 s and 40 cycles of 95 °C for 5 s and 60 °C for 30 s. Relative gene expression levels were calculated using the 2^−ΔΔ*C*t^ method [54], normalized using log2 transformation, and depicted in a heat map using HemI 1.0 software.

## 5. Conclusions

In this study, we conducted a systematic analysis of the SBP-box gene family in tea plants. A total of 20 *CsSBP* genes were identified and classified into six groups based on phylogenetic relationships. Gene structures and conserved motifs were also examined. The expression patterns of *CsSBP* genes in different tissues suggested potential functions in tea plant growth and development. In addition, the *cis*-elements and expression profiles of the *CsSBP* genes were examined and showed the important roles of *CsSBP* genes in regulating responses to abiotic stress and hormones. This work provides a solid foundation for further investigating the SBP-mediated molecular mechanisms underlying physiological developmental processes and stress responses.

## Figures and Tables

**Table 1:** The 20 *CsSBP* genes identified in tea plants and their sequence characteristics.

Name	Gene ID	Genomic (bp)	Exons	CDS (bp)	ORF (aa)	MW (kD)	pI
*CsSBP1*	CSA019508	2758	3	1050	349	38.22	7.64
*CsSBP2*	CSA020439	4000	3	1167	388	43.02	8.64
*CsSBP3*	CSA007311	3035	3	1521	506	55.63	7.06
*CsSBP4*	CSA031667	6349	4	2136	711	77.83	8.40
*CsSBP5*	CSA023442	7229	3	1221	406	44.81	6.58
*CsSBP6*	CSA013148	2239	4	1074	357	39.22	8.97
*CsSBP7*	CSA011373	5594	4	1440	479	52.63	8.23
*CsSBP8*	CSA013351	4629	12	3399	1132	124.64	8.29
*CsSBP9*	CSA015286	4622	10	3150	1049	116.38	8.01
*CsSBP10*	CSA034050	2108	4	933	310	34.73	9.02
*CsSBP11*	CSA034053	1117	2	810	269	30.03	8.69
*CsSBP12*	CSA034591	6500	10	3147	1048	11.60	6.54
*CsSBP13*	CSA025811	9742	11	2472	823	92.58	5.60
*CsSBP14*	CSA000458	3439	9	1527	508	56.89	6.45
*CsSBP15*	CSA022307	1789	2	450	149	16.95	6.19
*CsSBP16*	CSA036632	373	2	333	110	12.39	6.09
*CsSBP17*	CSA009667	1991	2	558	185	20.90	9.05
*CsSBP18*	CSA031785	3317	4	627	208	23.06	9.12
*CsSBP19*	CSA034418	6946	4	873	290	32.59	9.14
*CsSBP20*	CSA020672	621	1	618	205	22.77	6.41

## Supplementary Materials

Supplementary materials can be found at http://www.mdpi.com/1422-0067/19/11/3404/s1.

## Abbreviations

SBP	*SQUAMOSA* promoter binding protein
ABA	abscisic acid
GA	gibberellic acid
MeJA	methyl jasmonate
TFs	transcription factors
NLS	nuclear localization signal
UTR	untranslated region
FPKM	fragments per kilobase million
SOS	salt overly sensitive
ROS	reactive oxygen species
JA	jasmonic acid
SOD	superoxide dismutase
POD	peroxidase
SA	salicylic acid
