# Supplementary material for: Identification and Expression Analyses of SBP-Box Genes Reveal Their Involvement in Abiotic Stress and Hormone Response in Tea Plant (Camellia sinensis)
Source: Int J Mol Sci. 2018 Oct 30;19(11):3404. doi: 10.3390/ijms19113404 (PMC6274802; doi:10.3390/ijms19113404)
Supplement: Supplementary file 1 [file ijms-19-03404-s001.zip › Supplementary Materials/S1 Table.doc]

**Table S1. Full-length protein sequence of SBP genes from tea plant.**

>CsSBP1

MLGRMGGPADWSFNRIEDPKVSLMESSSSSGSSKRAKAPSTTSHVVSCLVDGCSSDLSQCREYHRRHKVCERHSKTPKVTIGGQEQRFCQQCSRFHSLVEFDEGKRSCRKRLEGHNRRRRKPQSESLSRNPGRFLSNQQGPTLLSFSSSQLFGSPMMNSAWAETVNAENSTLPYNSNQCLNYIDRHDNFLGSLPHSFGGGNQFKIFHGNNLPDLSEASTCQPLLDPSSASGTSSHSQKIMFSDGLSRAIVESDCALSLLSSAPPAETREIGLGHVVQPNPGSSAQSLAHNMHYCDLSQYSQGIESKPLASFLSSHAGDNADLHRPGMFQDGPDGPSDSGPHQTLSFRWE*

>CsSBP2

MDWELKASGWDFTDFGREDDTRLATLVGSSSLGVHKNGRGFSVDLKLGGLGDLGDGSIDKLKEPGGSTIASSPSGSLKRTRAINGTQNVSCLVDGCIADLSNCREYHRRHRVCERHSKTPVVIVGGKEQRFCQQCSRFQSLGEFDEEKRSCRKRLDGHNRRRRKPQPEAFYMSSGSFLSNPQGVRLLNFTGPQAYTTSTTGAVPTWPRVSEYHQDLHVVNQQSTTPNTLGCVYNGGDKRFPFLLANEPERSSNQAVPEFSICQRLFNDRASPERGRGFQKIPSDGLAQSVNSKLALSLLSSNRTQNSETSLTHILQRDMIHPNQPMGMSFQFDGLAQYSRSQGVVEEKPPVLVPQGGNTNNLCNEIFQVAPYDFLEEGASQVIPFSWK*

>CsSBP3

MESWSYVSQGKGFVSDDSISAPDVFGRGKNGLMGWELKTPCNYDSNLEENQGFVELGFPEMPRKSWPENNLNERICTPITSMTNAFSGEDESSSKFSSSVVESNSRDSSFIDLKLGRIADDFRSSQTAPVLSSSESLLPTKRVRAGLSSQTPFCQVYGCKKDLSSSKDYHKRHKVCEVHAKTAKVIVNGNEQRFHSLAEFDDGKRSCRKRLAGHNERRRKPHVGTHSSRSGRFFQSYNGSMITPSSFICQDILPSNILHPQKYDTNDWNGHIKLENGANYSPPSAIPITNGHLHPKSLFPHGFEKHCPFLANGVNSATGRDFNENSNRYTHYLGSSNSVSGSLIHNTSIGSEDFALFNSGSTVQGIGMSGCGRALSLLSSHSQNSSSHSSGIPMAQIVSGSHANYNVGQISGKILGVSSQASINGVTANKFNSVEENHFNPILISDDGDAVNFTDGMFQGSQYINDKDRLSCEDGSTIDLLKLSSQLQRVEHQRQFMHVKRENDAF*

>CsSBP4

MMFIQRMESWRHALEEKSPFLCDEKVSVGWNMKPSCNFDSMLGSNREAIEGMEFVEMGFPDMMRKLDPMNSCLDALNGEIGDYFSRTLHSPAYRNASNSSFGELDPGLSLSSPVIVSNTQKSSLIDLKLGRLGDCKEAKNSIFSNEISVLSSSGSSLKSKRARLENLSSQNPVCQVHGCNKDLSSSKSYHKRHKVCDIHSKTARVIVNSIEQRFCQQCSRLTILIENKLFLIPGFICWLNLMMENAVVENALQATMSVEESLNWILSLVAISTLSSSVCHVTQPSRPPWKPIINSGEAPRTRPMHLVNSSPSATAYSIGMPLMHTPASPATARAKRVFPVPGGLLWFHFRLCSSVSPRAS

LPQAQVLTNNNHKNPCLLSATLFSGKHTPNPALFSLSTPNQSFVIDGRSLSTATMNNGSTHQAGPSYWDPPPPPVTTAAGNSEKWGTHIMGAPAVPTSHLANQKAALWITGGARFLRTPLPKRSSFVFPDMLPGGYFSSEGNAQGNQSNHIKVESEPIYSPQLSMLTTNRQSHSKPVFHLHSMGKQNASEKISSQLGYTFLETELSGISNSSIALSLLSDQTKTLSSNLTGIPMLSPLITQGTNAHCGINPNSEDFVVSPNGFHSSGMNSMELDRRGPSVIFDAGIDIDFEVEAGGIFQRSDSVKSKSCLSHEHGHVDLIQLASHFQRVEQQRNSMQVKQENSVFCSFPTM*

>CsSBP5

MENGWSLFNTSITATTNTSTTNNSTTTTSSSSRGHNGHCNNLAWDIWDMGTSRFDWNTTSSTSFLHHHHQQQHQQQPPSSATVTAASEAHALMLGHQTAASLYSGAAGPHYYHPDPHLMCLKLGKRHYFEDATGAPLGDCHVVGGGGGGGKRGKTYYGGGGGGEVVGTSAPAPRCQVDGCNVALVNAKEYHRRHKVCEMHSKAPKVEVLGLEQRFCQQCSRFHEVSEFDDSKRSCRRRLAGHNERRRKGSHDSIPRSPHPSLSHGRALSLLSSSWKNNSWFSSSDLSLRCSAALRELIAENRAAILARQLVLERDWHRGSHDTMDDVNATQLAGTNPMTPHHHQMFPEPHGYWDRFNEGSGHVTLDLMQAPSSAFGLLSVRGKSKEEEEEEEEEEEECSGLWTHMV*

>CsSBP6

MDWNAKWDWENLITFNSKTVESPKKQPTDWEIGEGEIDAVSFNLSGGGAVGSSSDLAHGSSARSSVSASMDSSSKEISKMSKIAVEAFHDFPEDFGKKNELARTELAGTSLPLEPSVGSGELLIGLKLGKRTYFENNYAGDNAKTSSLSVIPVSSAASTAKKIKSSSQTHRQRCQVEGCNLDLSSAKEYHRKHRVCESHSKCAKVVVCGLERRFCQQCSRFHNLSEFDEKKRSCRKRLSDHNARRRKPQQDAIQFDSSMLLSSPFLDGKQQMCFALNNTPLLRTRSSANPTWESACSSKYFTLTKGHLSRSGNTGGIDGQPQLPGIELPHSISMLGQNTRAEIFTRGNSALNVVEIV*

>CsSBP7

MNSLPAMDWNSKWDWENLVMFNSKSIESPKKQPTDWGIEEGEIDMVSFNLSGGGGGGSGSELAHGSSARSSISASTDSSSKEVMKASRNAFEVFHGFTGDFSKKNELARAELAGTSPPLESSVGSGEPLIGLKLGKRTYFENNCTGGNTKTSSFSAIPASSAATTAKKIKSSCQSTPPPRCQVEGCSLDLSSAKDYHRKHRVCESHSKCPKVVVGGLDRRFCQQCSRFHSLSEFDEKKRSCRRRLSDHNARRRKPQQDVNQFNSTRLPSSFLGIDFYLDFEPVASYGRQQMSFVLNNAPLVHTRSSANPTWESTCNPKFTLTEGYLLKSEKNGGIDGQLQLRGTELPHAISMHGQNSNRLFPSKGSKAEIFTQGLSSNLDAAPDHRALSLLSTNSWGSEPANIALDHPIHVNLTTMPQPMIHGVPQGLPLSSSDYWQTEQHCTDPRHIHPLTTNDGSQFHEIQLLKSPYESALYPNLMN*

>CsSBP8

MEEVGAQVSPPIFIHQTLGGDRFREANPMATKRSLPFHAPNFQQQQPGLTSGYNWNAKDWVWDSVSFIAKPMESGVLSLATATSVQPQSRPGQHRKSDREGSGLPTLLKKNPVNEDDESLRLKLGAGLNSFEESVSRPNKRVRSGSPGSGGGGSGSGSYPMCQVDNCREDLSNAKDYHRRHKVCEVHSKSSKALVGKEMQRFCQQCSRFHPLTEFDEGKRSCRRRLAGHNRRRRKTQTEDVSSSMLLPANHDTSGNGDLDIVNLLTVLAHAQGNKEVKSSNGSSLPNKDQLIQILSKINSLPLPVEFEAKLPPVSGSSSRMTIPEQASSDNQSSGLNGNTTSPSTKDLLTVLSTTLAESSPDALAILSQRSSQGADVDETKLACLDQALSLNMLKGASLEFPSVGGERSSTSSQSPVDYSDSQIQETRAHLPLQLISSSPEDDSLPKLASARKYFSSTSSNPTDETSPLSSPPVVQKLFPMQTTRETEPARITTNGEVNKNVETSNARGCVTSLELFGRSNISANNGSFKSFPYKAGYTSSSGSDQSPSSSNSDAQDRTGRIIFKLFGKNPSQFPESLRTEIYNWLSHSPSEMESYIRPGCVVLSIYLSMPSSAWGQVALTLTICKGCLLEENLLRHINSLIRDSDTDFWRDGRQANLLDWCYQSSGESCHCKSWKAWRSPELISVSPLAIVGGEETSLFLRGRNLNIPGTKIHCTHMGG

YLSKEVPGSSGRETLYGEISVVNFKVHGVAPGVLGRCFIEVENGFKGTSFPVIIADATICQELRLLESEFNEGAKVYDTISEDQIHDLGKPRSKEEVLHFLNELGWLFQRKRNSSAIEVRDYTLARFKFLFIFSVERDCCALVKTLLDILFERYLERGEPSRESLEMLSEIHLLNRAVKRRCRNMVDLLIHYSVLSGNDTSKYIFPPNLVGPGGVTPLHQAACTSSSDVMVDALTSDPQEPIPECLLNFMDAPKKRYNYSRLHEASVSPKIGLYCWTSLLDANGLSPYAYALTRNNHSYNRLVARKLADRRNSQVSVSIGNEIDQQRLGASLDHRQRSQFKQGPSSCSKCAVVASRYSRR

MPGSRGFIHLPFMHSMLAIAAVCVCVCLFLRGRPDVGLVAPFEWESLGCGSS*

>CsSBP9

MEEVGAQISPSIFIHQPLPAQFCEGHPNPMAKKRNLSFQVQQQLASGFQIPGNIWKPKDWNWDCVRFAAKPLKSDVLHLGTSQLVHSEQHKIGRESSGISTLLEKNPVDQDNEILQLQLGVSLNSLQECVSRPNKRVRSGSPGGGGGGNGSYPVCQVDNCREDLSKAKDYHRRHKVCEIHSKSSTALVGKQIQRFHHLSEFDEGKRSCRRRLAGHNRRRRKTQTEDVSSRMLLPGSHDNAGNGDLDIVNLLTVLARAQGNEEVNSPNCSSVPNKDQLIQILSKMNSLPLPVDFETKLPVSESLTSTVPEQSFSDYQNKLNRNMSSASTKDLLAVLSATLAASTPDALAILSQRSSQGVEADKTKLACLEQALSLNMQRATAFEFPSVGGESSNTSYQYPVEDSDCQVQEIRANLPLQLFSSSPEGDSLPKLTSARKYFSSGSSNPMEETSPSSSPPVVQKLFPRQTTRETELERISTIQEVKENVEISAAHGCVSSLELFGGSNRGADSGSFQTLPYQAGYTSSSGSDHSPSGLNSEAQDRTGRIIFKLFGKDPSQLPGTLRTQVYNWLSHSPSEMESYIRPGCVVLSIYISMSSSSWEQALHYTIQISQKEEKYGKIRLCKSWTAWRPPELISVSPLAVVGGQETSLFLRGRNLNIPGTKIHCTHGYLSKEVPGSACQETIYDERSLVRFKINGVGPGVLGRCFIEVENGFKGTSFPVI

IADANICQELRLLESELDEAAKIHDTISENQICNFGRPGSKEEVLHFLNELGWLFQRKQNSSTIEVPDYTLTRFKFLFIFTVERDFCALVRTLLDMLLQRDLDRGEPSRESLEILSEICLLNRAVKRRCRNMVELLIHYSVLSGNDTFKYIFPPNLVGPGGITPLHLAACTSSSDDMVDALTSDPQEIGLHCWNSLLDANGLSPYAYALMRNNHSSNRLVTRKLADRKNGQVSVSIGNEIEQQCLRVNREHQQRSQFKRGQRSCSNCAVVATRYNSRIAVSQGLLHRPYMHSVLAIAAVCVCVCLFMRGHPNVNLVNPFKWENLGYGPM*

>CsSBP10

MEAQFGGKAYHLYGPVIPNLKAVGKKSMEWDLNDWKWDGDLLTAAPLNSVPSDCRSRQLFPVVSEIPINTALSKSSSSCSEETNLGNEKGKRALEKRRRVVVVEDEEFNDGAGSLNLKLGGQVYPITEAEMEKSVGKCGKKSKVVASTSNRAVCQVEDCQVDLTNAKDYHRRHKVCDMHSKATRALVGNVMQRFCQQCSRFHVLQEFDEGKRSCRRRLAGHNKRRRKTHPENVVSGGSLNDEHGSSYLLISLLRILSNMHISFDLIIKYVEVHNENVKTVMCNLYNSYVSLWEPNFIGSIGTNGRVQSGT*

>CsSBP11

MEEAQFGGKVHHLCGPVMPDLKAAGKKGVEWDLNDWKWDGDLFTAAPPLNSVPSGCRSRQLFSLVSEIPINPGLSNTSLACSDETNLGNDKGKRVLEKRRRVVVVEDEEECNDEARSLNLKLGGQVYPITQAEMENSEGRCGKKSKVVATSSNCSVCQVDDCRVDLTNSKDYHIRHKVCDMHSKAGSALVGNVMQRFCQQCSRFHVLQEFDEGKRSCRRRLAGHNKRRRKKHPENVVNGVSLNDEHGSSYLLVSLLRILANMHSKYLSS*

>CsSBP12

MEARFGRKAHHFYGPVMSDLKAVGKKSMEWDLNDWKWDGDLFTATPLNSVPSDCRSKQLFPDGSEIPTNTGLSNSSSSCSDEINLGNDKGKRELEKRRRVAVVEDEELNDGGGSLNLKLGGQVYSITEAEMEKWEGKSGKKSKVAAAATSNRAVCQVDDCRSDLTNAKDYHRRHKVCDMHSKASKALVGNVMQRFCQQCSRYNLVIPMGRFHVLQEFDEGKRSCRRRLAGHNKRRRKTHPENVVNGGSLNDEHGSSYLLVSLLRILANTHTNSTDQTKDQDLLSHLLRNLANLAGSINGNNISGLLPGSQNLQNAGTSLGTPEKVSNLISNSPKHPSIFGSTSNTDDFIDIQDPPKLNGQCTTVSVLEGIEKRTFSDDPLGGTLQTPSTILCPTRDSIPAIASGPETAAGRMKLNDIDLNNVYDDSQDCAENLDSYHAPVNPGTGSLDCTVWGHQHCQKSSPPQTSGNSGSMSTLSPSSSSGETQSRTDRIVFKLFGKDPSDFPLVLRKQILDWLSHSPTDIESYIRPGCTILTIYLRLGKSTWDELCCDLNSSLTSLLDASNDSFWRTGWVYARVQHRVAFTYNGQVVLDTLLPRKSHKNCRISSIKPIAVSISKRAQFLVEGFNLYQSTTRLLCALEGKYLVQESCSDMMEGGNTSIDHDEIQCLSFSCSIPNVTGRGFIEVEDHGLSSSFFPFIVAEHDVCSEICMLESEIGVAEIADDIQAESEQMNARNNALDFIHEMGWLLHRCQLQFRLGSADPNLDLFPFKRFRWLMEFSLEHNWCAVVKKLLRISFNGTVDAGDHSSIELALLEMALLHRAVHKNCRSMVEMLLRYVPDGVLDKPDSEQKQHVYSSSNFLFKPDAIGPGGLTPLHIAASRDGSENVLDALTDDPGLVGINAWKGTHDSTGLTPNDYACLRGYYSYVNLVQKKINKKKSGNEHVVLDIPGALLAHNIKQKLMDGHRSAKVASLHTENGEMKPIQQHCKLCEQKMAYRNPRASLAIYKPAMLSMVAIAAVCVCVALLFKSSPEVHCVFQPFRWELLKYGSS*

>CsSBP13

MDTLSLPSPPSPPPPPQPSVSDMENLFSNPPLNDDPSSSSLWDWSDFLDFNLEDQLNISFDSSENTPVLPPPDSDSVDRIRKRDPRLTCSNFLARIPCACPELDEKEEEEEGVPVKKRPRAAAGAAARCQVPGCEADISELKGYHRRHRVCLRCANASTVVLDGLSKRYCQQCGKFHILSDFDEGKRSCRRKLERHNNRRRRKPSESKVAVEKDPQELVLADDVACDDENEKDSTCLSSQLTEREPLLESEEGQIPTLCSNPSSQNIQSDSVASFVVSREPQIDGEKDESKHAHSSFYCDNKSAYSSLCPTGRISFKLYDWNPAEFPRRLRHQIFQWLSSMPVELEGYIRPGCTILTAFIAMPNSMWFKGFHDLTSLLHSVEQLLQEPVVYIHDFVAAPGKMLSGRGTFLVYLDNVIFRVMKDGTSVIQIKAEEQAPKLHYVHPTCFEAGKPMEFVACGSNLLQPKFRFLVSFAGKYLAYDYSVPPPYDDSEGVTANFNHQLLKICIRHTEPDLFGPAFIEVENESGLSNFIPILIGNKEICSEMKIMEQRFEASLSLSLKESKFMAASSVHDSCEVSILRQTAFSQFILDTAWLLRNPALEDMKYRLSSSQIGSFNRLLTFLIQNKSISILERVLHYMETVMDDKKLHCMTNGIADAEMKLLQSNVDHARGIVSARLQENVDSVRHSRNLVPKGNCVYQSSQIDMRSVVSFIDQNMEVTEKGSLDAVEGSNSLDHSGTVPLLNRDVEMSMKEWPRKSCSLVFSSRILTSRPLIFVIVAAAVCCGICVVLLHPQKVGKIATTIRRQDDLNFQVASYQQNQDTV*

>CsSBP14

MDSPPPPPPPLPPPHSPQPRVSEPENPIPHDLWDWSDLLDFNLDINQLNSSLDPVQPSPDPQFPPTDPNLDIPGRVRKRDPRLICSNFMAGRIPCACPELDEKLEEEERLQPGKKKARTVRPAAGGKAKANARCQVVGCEADISELKGYHKRHRVCLQCANASDVGIDGQRKRYCQQCGKGSNDPTTLHVVPPMLKGKRSCRRKLERHNNRRRRKASEATVAVEREPQEPVLVDDVACDDENGKVVELKEFCGSDSICLSSQIAERESLLESEEGQISTLCSNPGSQNIQNDSGISFVASGEVQINGEKDSKHAHSSFYCDNKSAYSSTCPTGRISFKLYDWNPAEFPRRLRHQWLASMPVELEGYIRPGCTILTMFISMPNFMWSKLHEDPVMYIPDLVGAPGKMLYGRGSLLVYLNNVIFRVMRDGTSVMQVKVEEQAPKLCYVHPTCFEAGKPMEFMACGINLLQPKFRKGEQAVAYGTQTPLEEQLKQEGSCKYKDFPTCDAWN*

>CsSBP15

METGKAGGEKSVKDKMKGEVVEEEEEEEEDEDEDDVGLGSGEDDKKKKEYNKKGSSRGGSTLPSCQVDNCTTDMTYARPYHRRHKVCEFHAKLAVAVVAGLRQRFCQQCSRFHELSEFDETKRSCRRRLIGHNERRRKSSHDTHGEGSS*

>CsSBP16

METIKAGGKRRMKEVEGDEEEEEEDEDVVELDSGEDNKKMKGPSGRKGSSSGGSRQRCCQVDNCTTDMTYARSYHRRHKVCEFHAKAPAVDVAGLHQRFCQQCSRSQLSM*

>CsSBP17

MEFGKQRGGMNMMKDKVKMEAAADGDDFDKDDNNDDFVNASKDDKKKRASGGGGGGSSMRICQAENCTTDLADAKQYHRRHKVCEHHAKAQVVMVAGIRQRFCQQCSRFHELSEFDEAKRSCRRRLAGHNERRRKNTSDSHAEGSSHKGTGTHQLNENASGQVDERGRIQISIQENATYKHFHIR*

>CsSBP18

MDSRRPNPSSLSKQKSDDFAPDDDDDSVEEEDENYASEFGISSEKGGLKKKNKKKKVKVGFDGGSGKKRGGGGGGLLSSRPRCQAEKCEADLTEAKRYYRRHKVCELHAKATAAIVAGLRQRFCQQCSSMMPFNGERNFSSNPASFMSYLSLMTQREVAEGVWRGIMSDAGRAHRNHPMEEEKKGVVAKDWAIKYIIKLGHDLLLVVG*

>CsSBP19

MLEYEWGNPSTVIFSAEDSDQSRQIFDQSFNETLLHQTGFSQPHYHFHQNPTQTQTQTHTHSHFHSLYDPRSLFAPSYPPPSPAFLPPVAGPTGFVPKSEPGGGVVDFGSRIGLNLGGRTYFASSSGDDFVNRMYRRPRSVEPGSMINSPRCQAEGCIADLTHSKHYHRRHKVCEFHSKAATVVTAGLTQRFCQQCSRFHQLSEFDNGKRSCRKRLADHNRRRRKSHHQLPNHETKSQLQNARHSSSENLARTSANSEAQSSSLVTMAISPPRISLDCNVKSQAPSTMDD*

>CsSBP20

MLEYEWGNPSTVMHSGEEPTGDSDPNRQIFDHYAQAFNETLVNSTGFSQNQHHFHQPQQPFHHPQTQAYSHSLYDPRAFGVSYAPPSPAMLSLDHVGGATVEHGGYVVAPKSEPVADFASRIGLNLGGRTYFSSSEDDFVNRLYRRSRAVEPGSVNSPRCQAEGCNADLTHAKHYHRRHKVCEFHSKASTVIAAGLTQRFCQQSG*
